# Machine learning model for predicting the optimal depth of tracheal tube insertion in pediatric patients: A retrospective cohort study

**DOI:** 10.1371/journal.pone.0257069

**Published:** 2021-09-02

**Authors:** Jae-Geum Shim, Kyoung-Ho Ryu, Sung Hyun Lee, Eun-Ah Cho, Sungho Lee, Jin Hee Ahn

**Affiliations:** Department of Anesthesiology and Pain Medicine, Kangbuk Samsung Hospital, Sungkyunkwan University School of Medicine, Seoul, Korea; Qingdao University, CHINA

## Abstract

**Objective:**

To construct a prediction model for optimal tracheal tube depth in pediatric patients using machine learning.

**Methods:**

Pediatric patients aged <7 years who received post-operative ventilation after undergoing surgery between January 2015 and December 2018 were investigated in this retrospective study. The optimal location of the tracheal tube was defined as the median of the distance between the upper margin of the first thoracic(T1) vertebral body and the lower margin of the third thoracic(T3) vertebral body. We applied four machine learning models: random forest, elastic net, support vector machine, and artificial neural network and compared their prediction accuracy to three formula-based methods, which were based on age, height, and tracheal tube internal diameter(ID).

**Results:**

For each method, the percentage with optimal tracheal tube depth predictions in the test set was calculated as follows: 79.0 (95% confidence interval [CI], 73.5 to 83.6) for random forest, 77.4 (95% CI, 71.8 to 82.2; *P = 0*.*719*) for elastic net, 77.0 (95% CI, 71.4 to 81.8; *P = 0*.*486*) for support vector machine, 76.6 (95% CI, 71.0 to 81.5; *P = 1*.*0*) for artificial neural network, 66.9 (95% CI, 60.9 to 72.5; *P < 0*.*001*) for the age-based formula, 58.5 (95% CI, 52.3 to 64.4; P< 0.001) for the tube ID-based formula, and 44.4 (95% CI, 38.3 to 50.6; *P < 0*.*001*) for the height-based formula.

**Conclusions:**

In this study, the machine learning models predicted the optimal tracheal tube tip location for pediatric patients more accurately than the formula-based methods. Machine learning models using biometric variables may help clinicians make decisions regarding optimal tracheal tube depth in pediatric patients.

## Introduction

Pediatric patients have a shorter tracheal length than adults; therefore, the safety margin for tracheal tube tip positioning is narrow. However, the tracheal tube tip is misplaced in 35%–50% of pediatric patients and can cause hypoxia, atelectasis, hypercarbia, pneumothorax, and even death [1–4]. Therefore, in pediatric patients who require mechanical ventilation, it is crucial to ensure the tracheal tube is placed at an optimum depth.

Chest X-ray is the gold standard method of confirming the placement of the tracheal tube tip [5]. However, since this verification method is time consuming and increases the risk of radiation exposure, a reliable, safe means of predicting optimal tube depth is needed [6]. Various methods have been proposed to estimate the optimal position of the tracheal tube tip based on age [7, 8], height [9], and the tracheal tube internal diameter (ID) [10]. However, due to a lack of accuracy, most of these estimation methods are not acceptable for clinical practice [11].

Machine learning, a subset of artificial intelligence, involves computer systems that learn from data, identify patterns, and can make decisions without following explicit programmed instructions. After giving training data to a learning algorithm, the machine learning model generates a set of rules which can also be used to make predictions for novel datasets [12]. Recently, several institutions have conducted airway management studies using machine learning and virtual reality, including a study by Xiao et al., in which machine learning and virtual reality were used to assess neonatal intubation [13]. Additionally, in a study conducted by Lakhani, deep learning was used to determine the appropriateness of the tracheal tube position using radiographic imaging [14].

Therefore, this study aimed to apply a machine learning method to predict the appropriate tracheal tube tip location in pediatric patients through constructing prediction models that estimated the optimal tracheal tube depth. The primary objective was to construct new prediction models using machine learning methods to estimate the optimal tracheal tube depth, and the secondary objective was to compare the machine learning models with the commonly used formula-based methods, which are based on age, height, and tracheal tube internal diameter.

## Methods

Pediatric patients aged < 7 years who received post-operative mechanical ventilation after undergoing surgery at Samsung Medical Center between January 2015 and December 2018 were investigated in this retrospective study. The exclusion criteria were as follows: vertebral column abnormalities, congenital abnormalities of the tracheobronchial tree, preoperative tracheostomies, and chest X-rays of insufficient quality. Ethical approval for this study (Samsung Medical Center Institutional Review Board SMC 2019-02-085) was provided by the SMC IRB of SungKyunkwan University Hospital, Seoul, Korea (chairperson prof. Suk-Koo Lee) on 4 March 2019. The need for written informed consent was waived by the SMC IRB due to its retrospective nature.

**Fig 1:**
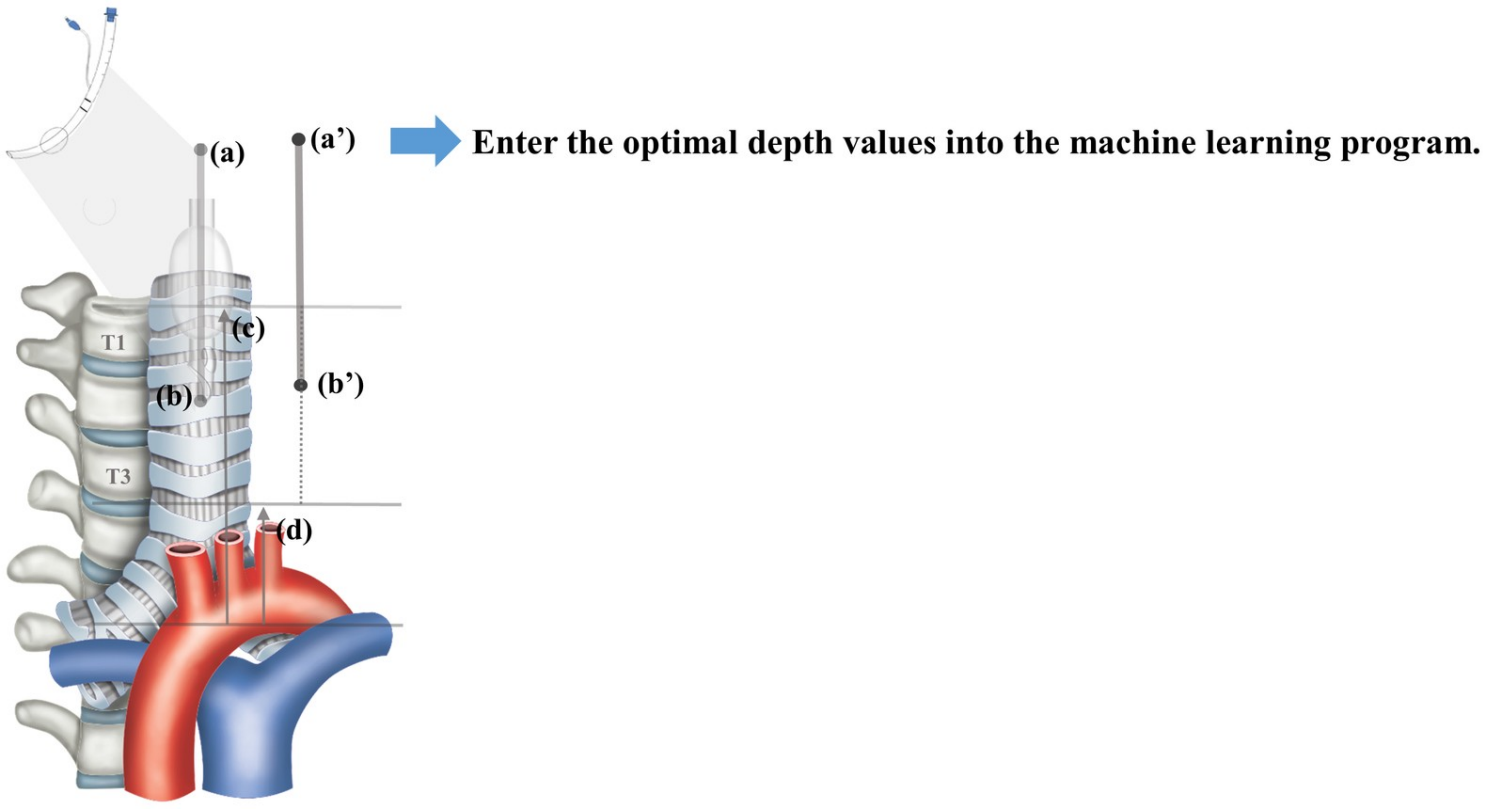
Method of estimating the optimal depth of the tracheal tube. **Step 1**. From the electronic medical record, retrieve the recorded depth of the tracheal tube (a). **Step 2**. Measure the distance from the carina to the tracheal tube tip (b) on the chest X-ray. **Step 3**. Assume that the median of the distance from the carina to the upper margin of T1 (c) and the distance from the carina to the lower margin of T3 (d) is the optimal tracheal tube tip position (b’). **Step 4**. The optimal tube depth (a’) is determined by moving the tube from the previous tube depth (a) to the difference between the tracheal tube tip (b) and the optimal tracheal tube tip (b’). **Step 5**. Enter the optimal depth values (a’) into the machine learning program.

**Fig 2:**
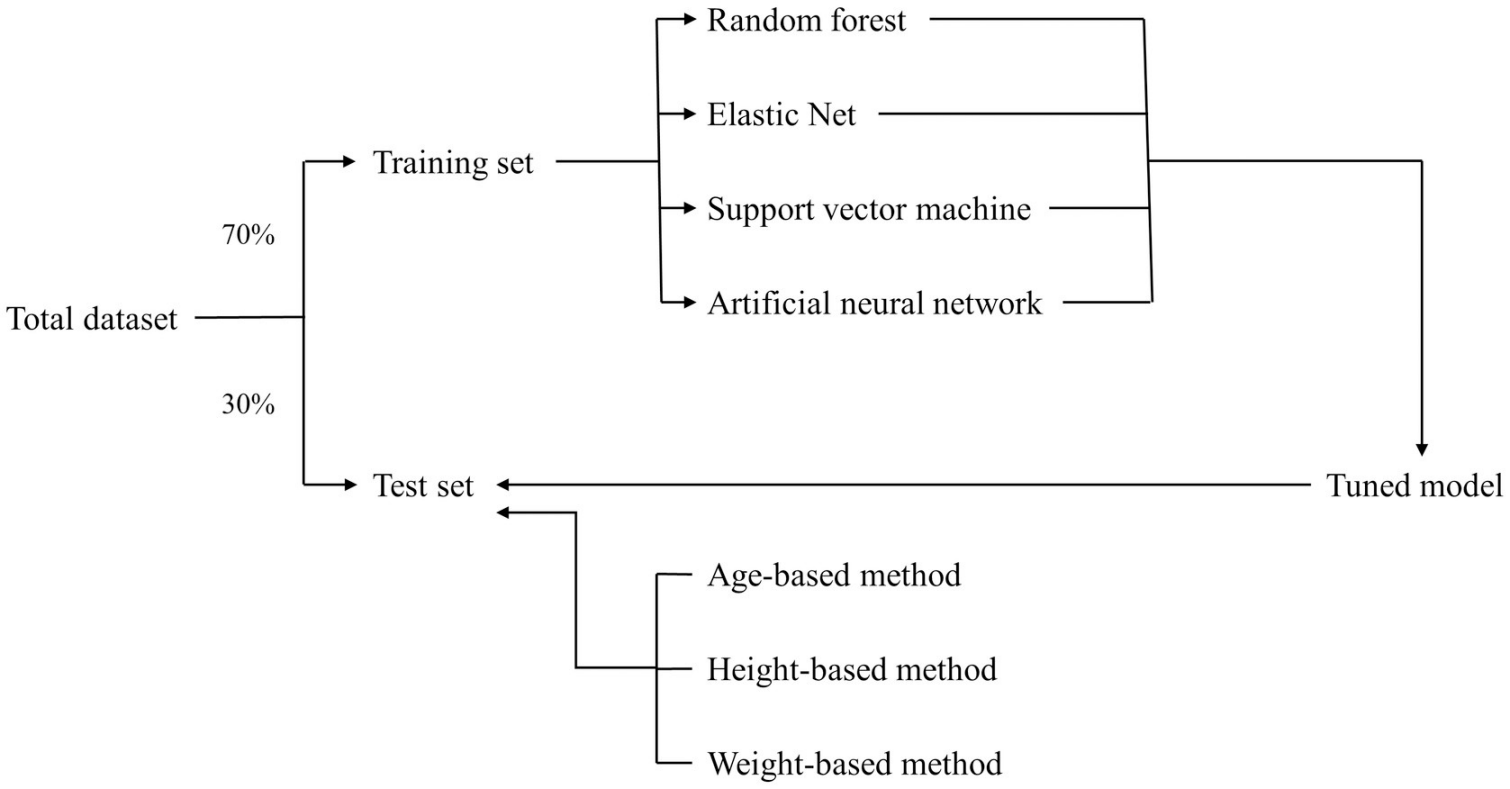
Flow diagram of application the machine learning and formula-based methods.

To develop machine learning models, the patients’ clinical data, such as age, sex, height, weight, and fixed depth of the tracheal tube were retrieved from the electronic medical record. Post-operative X-ray data were obtained using the INFINITT picture archiving communication system (INFINITT Healthcare Co., Seoul, Korea). The caliper function was used to determine the following: the distance from the carina to the upper margin of the first thoracic vertebral body (T1), the distance from the carina to the lower margin of the third thoracic vertebral body (T3), and the distance from the carina to the tip of the tracheal tube. Using the fixed depth of the tracheal tube obtained from the electronic medical record and the distance from the carina to the tip of the tracheal tube, the depth of tracheal tube was estimated when the tracheal tube tip was located at the T1 upper margin and T3 lower margin (Fig 1). Feature variables for the machine learning model included age, sex, height, weight, and the optimal depth of the tracheal tube. We set the target variable in training set as the median between the upper margin of T1 and the lower margin of T3, and entered that value into the machine learning program [15–17]. After dividing the total dataset into either the training or the test set, a machine learning model was developed using the training set and validated by the test set, as shown in Fig 2.

We applied four machine learning models: random forest, elastic net, support vector machine and artificial neural network model. Random forest is an ensemble learning method that consists of a large number of individual decision trees [18]. It is a bootstrap aggregating technique, so all calculations are run in parallel and there is no interaction between the decision trees when building them. Support vector machine works by identifying the optimal decision boundary that splits data form different categories [19]. The algorithm finds a line or a hyperplane which separates the data into categories. New input values are then predicted to belong to a category based on which side of the hyperplane they fall. Artificial neural network is a computational model that can be viewed as analogous to human nervous system [20]. Just like human nervous system, which is made up of interconnected neurons, a neural network is made up of interconnected information processing units called perceptrons. In fact, artificial neural network has the advantage of parallel processing of information, which allows it to deal with non-linearity. Elastic Net produces a regression model that linearly combines the L1 and L2 regularizations of the lasso and ridge methods. The consequence of this is to effectively shrink coefficients and to set some coefficients to zero.

For the age-based method, insertion depth was determined as follows: 9 cm for those aged 0–6 months, 10 cm for those aged 6–12 months, and 11 cm for those aged under 2 years [7]. For those aged ≥2 years, the correct tube depth was estimated using the pediatric advanced cardiac life support formula (tube depth [cm] = 12 + age [years] / 2) [8, 15]. For the height-based method, the tube depth was based on the Morgan and Steward formula (tube depth [cm] = height [cm] / 10 + 5) [9]. The insertion depth of the tracheal tube based on the tracheal tube ID was estimated using the formula: 3 x (tube ID) [10].

In machine learning tests, the determination of the appropriateness of the tracheal tube depth was defined as the case where the tracheal tube tip was located between the upper margin of T1 and the lower margin of T3 [5, 21]. The test set was run on the machine learning model (random forest [18], elastic net [22], support vector machine [19] and artificial neural network [23]) and the formula methods based on age, height, and tube ID were calculated. We also used 5-fold cross-validation which is reliable for relatively small data considering the size of our study. The optimal tube depth predictions for each of the machine learning models were compared to that for each of the formula-based methods.

### Statistical analysis

Continuous variables, including age, height, and weight, were presented as the median (interquartile range), while categorical variables, such as sex, were presented as numbers (%). R software (version 3.6.3) was used to construct the prediction models (using regression models) and to analyze our data. Before constructing the prediction models, the collected data were randomly divided into either the training or test set. Of the total datasets, 70% were used for training the machine learning models, and 30% were used as the test set for verification. To determine how well the regression models were able to predict tracheal tube depth, the percentage that predicted the optimal depth was calculated with a 95% confidence interval (CI). The missing data in weights were imputed using a nearest neighbor imputation algorithm, which identifies the neighboring point to fill in missing data through measuring distance [24]. The difference between the machine learning methods and the other methods was analyzed using the McNemar test, and significance was determined by p < 0.05. The programming code of our machine learning algorithm is freely available online (https://github.com/jgshim/tube-depth).

## Results

In this retrospective study, total 859 children were assessed. Among these, 17 patients who had tracheostomies and 8 patients who had insufficient chest X-ray quality were excluded. As a result, a total of 834 pediatric patients were enrolled in the study. The patients’ demographic and clinical characteristics are presented in Table 1.

**Table 1:** Patient demographic data and variable features.

Variables	Total, *n* = 834(100%)	Training set, *n* = 586(70%)	Test set, *n* = 248(30%)
Age (years)			
< 6 months	479	338	141
6 months–1 year	98	68	30
1–2 years	112	80	32
2–7 years	145	100	45
Height (cm)	63 [54 to 78]	63 [54 to 78]	63 [53 to 80]
Weight (kg)	6.2 [3.9 to 10.0]	6.2 [4.0 to 10.0]	6.1 [3.9 to 10.0]
Sex (female)	391 (46.9)	271 (46.2)	120 (48.4)
Tube size (ID in mm)	3.5 [3.5 to 4.0]	3.5 [3.5 to 4.0]	3.5 [3.5 to 4.0]
Distance from carina to T1 (cm)	3.2 [2.6 to 4.2]	3.2 [2.6 to 4.2]	3.3 [2.6 to 4.1]
Distance from carina to T3 (cm)	0.8 [0.4 to 1.3]	0.8 [0.4 to 1.3]	0.8 [0.5 to 1.2]
Distance from carina to tracheal tube tip (cm)	1.0 [0.6 to 1.5]	1.0 [0.6 to 1.5]	1.0 [0.6 to 1.5]

Distance from carina to T1 upper border, T3 lower border and tip of tracheal tube was measured using the postoperative chest X-ray.

The data are presented as median [interquartile range] or number (%).

ID, internal diameter.

**Fig 3:**
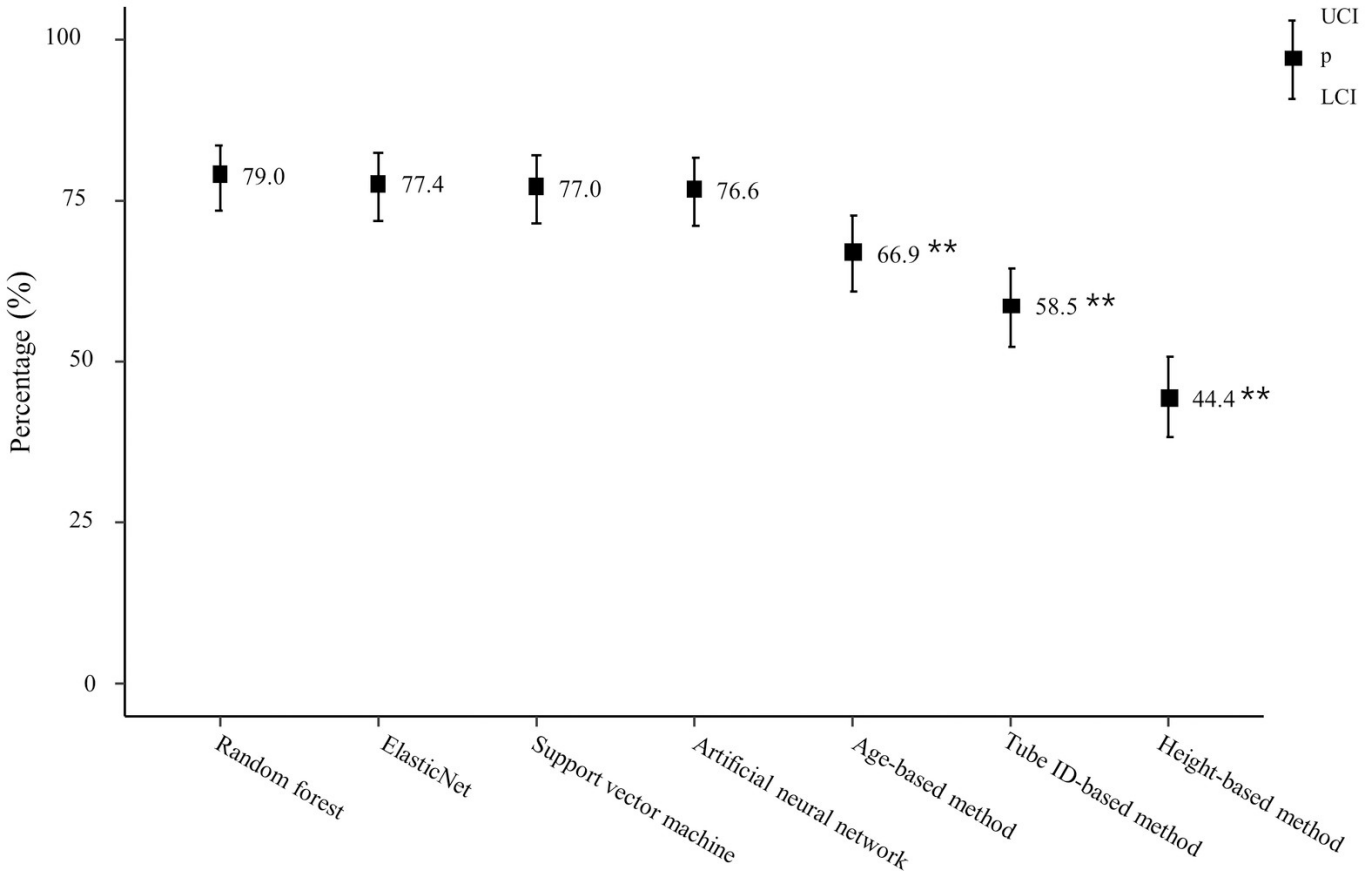
Percentage of tracheal tube tip positioned in optimal location. (Random forest method versus Age-based method or Tube ID-based method or Height-based method). Comparisons between the random forest and formula based methods (Age-based method, Tube ID-based method and Height-based method) were performed using the McNemar test, respectively. ** *p* < 0.001.

For each of the four machine learning algorithms and three formula-based methods, the percentage that predicted optimal tracheal tube insertion depth in the test set were shown in Table 2 and Fig 3. The percentage of located the optimal depth in machine learning models were: random forest, 79.0 (95% CI, 73.5 to 83.6); elastic net, 77.4 (95% CI, 71.8 to 82.2; p = 0.719); support vector machine, 77.0 (95% CI, 71.4 to 81.8; p = 0.486); artificial neural network and 76.6 (95% CI, 71 to 81.5; p = 1.0). Among the machine learning models, the random forest model had the most predictive power compared to other models, but, there was no statistically significant differences. The percentages that accurately predicted the optimal depth in machine learning models were: age-based formula, 66.9 (95% CI, 60.9 to 72.5; p < 0.001); tube ID-based formula, 58.5 (95% CI, 52.3 to 64.4; p < 0.001); and height-based formula, 44.4 (95% CI, 38.3 to 50.6; p < 0.001). When comparing the random forest model and the formula-based method, the random forest model was statistically significantly superior in predicting power (P<0.001, respectively).

**Table 2:** The percentage of optimal predictions for tracheal tube tip location for each prediction model (test data).

	Percentage of optimal predictions [95% CI]
**Machine learning method**	
Random forest	79.0 [73.5 to 83.6]
Elastic Net	77.4 [71.8 to 82.2]
Support vector machine	77.0 [71.4 to 81.8]
Artificial neural network	76.6 [71.0 to 81.5]
**Formula-based method**	
Age-based method	66.9 [60.9 to 72.5]
Tube ID-based method	58.5 [52.3 to 64.4]
Height-based method	44.4 [38.3 to 50.6]

CI, confidence interval; ID, internal diameter.

Data are presented as percentage and 95% CI (confidence interval).

Table 3 shows the predicted number of cases with deep or shallow intubation in both the machine learning method and the Formula-based method. The number of shallow or deep intubation cases was significantly higher in the formula-based method than in the machine learning method. Among formula-based methods, shallow intubation was observed more frequently in Age-based method (Shallow n = 55, 22.2% versus Deep n = 27, 10.9%), and deep intubation was observed more frequently in Tube ID-based method (Shallow n = 10, 4% versus Deep n = 93, 37.5%) and Height-based method (Shallow n = 7, 2.8% versus Deep n = 131, 52.8%).

**Table 3:** The number of cases predicted with deep or shallow intubation.

	Shallow intubation, n(%)	Deep intubation, n(%)
**Machine learning method**		
Random forest	31 (12.5)	22 (8.9)
Elastic Net	31 (12.5)	24 (9.7)
Support vector machine	31 (12.5)	26 (10.5)
Artificial neural network	33 (13.3)	21 (8.5)
**Formula-based method**		
Age-based method	55 (22.2)	27 (10.9)
Tube ID-based method	10 (4.0)	93 (37.5)
Height-based method	7 (2.8)	131 (52.8)

Data are presented number and percentage (%).

## Discussion

In this retrospective study, we developed and validated a new prediction model to estimate the endotracheal tube insertion depth for pediatric patients using a machine learning algorithm. To the best of our knowledge, this study is the first to apply machine learning to predict optimal tube depth in pediatric patients. Two important findings should be noted. First, the machine learning approaches outperformed the formula-based methods based on age, height, and tube ID. Second, there were no significant differences between the machine learning methods; however, the random forest method was the most accurate at predicting the optimal tracheal tube depth.

There are various formulas that can be used to predict the optimal tracheal tube depth using age, height, and tube ID; however, the accuracy of these methods is not reliable. While the gold standard method of confirming the tip of the tracheal tube is a chest X-ray [5], this method is time consuming and increases the risk of radiation exposure. A reliable and safe means of predicting the optimal tracheal tube depth is therefore needed [6]. Currently, effective methods to guide health professionals in real time when performing intubations are lacking [25]. Recently, a new prediction model, which analyzes computed tomography images, was proposed; however, it was only applicable to patients within a limited height range and individual differences were difficult to determine since only height or weight measures were used in the prediction equation [26]. Therefore, a prediction model that includes multiple variables is needed for clinical practice.

Our new prediction model has several advantages. First, the optimal tracheal tube depth can be determined considering multiple biometric variables (age, sex, height, and weight) rather than a single variable. Second, since we implemented a prediction model using the data that was actually applied to intubated pediatric patients, it can be applied safely in clinical practice. Third, compared to previous studies, a relatively large range of ages were used for the prediction models, as the entire pediatric population under the age of 7 who received ventilation during our study period were included. Unfortunately, it has not been implemented in this study, but there is a need not only to create machine learning models, but also to make it easy to apply in daily clinical practice. For example, create web or mobile applications powered by machine learning for easy access by users. By entering biometric variables (age, sex, height, and weight) into the mobile application, the optimal tube depth can be derived, it will provide useful information to clinicians unfamiliar with pediatric tracheal intubation.

Previous studies have also shown the advantages of using machine learning algorithms for prediction [27]. Lee et al. used a deep learning method to predict bispectral index during target-controlled infusion of propofol and remifentanil [28] and showed that the concordance correlation coefficient was 0.561 in the deep learning model, significantly larger than that in the response surface model (0.265). In addition, Shim et al. reported that machine learning methods could be used for predicting the occurrence of osteoporosis [29]. The artificial neural network used in that study had a better performance and lower error rates in osteoporosis risk assessment compared to the logistic regression model. In our study, however, the random forest model had the most accurate results, possibly because the number of datasets may have been insufficient to achieve optimal performance with the artificial neural network. Further research using more large-scale datasets are needed in the future to determine which machine learning algorithm most accurately predicts optimal tracheal tube depth.

Our study also had several limitations. First, our prediction model was based on patients of the same ethnic group who were hospitalized in a single center, so it may be difficult to generalize our prediction model to a diverse population. Second, the size of the study population was too small and therefore insufficient to verify the performance of the machine learning model, especially the artificial neural network. Since the performance of any machine learning algorithm is strongly influenced by the quality of the training data, larger and more diverse patient datasets are needed to increase the generalizability of the results.

## Conclusion

Machine learning model had more accurate predictive power than existing formula-based methods and can be used to predict the optimal depth of the tracheal tube in pediatric patients. Machine learning models using biometric variables may help clinicians make decisions regarding optimal tracheal tube depth in pediatric patients.
